# Maximum Target Coverage Problem in Mobile Wireless Sensor Networks

**DOI:** 10.3390/s21010184

**Published:** 2020-12-29

**Authors:** Dieyan Liang, Hong Shen, Lin Chen

**Affiliations:** School of Computer Science and Engineering, Sun Yat-Sen University, Guangzhou 510006, China; ldiey@mail2.sysu.edu.cn (D.L.); chenlin69@mail.sysu.edu.cn (L.C.)

**Keywords:** approximation algorithm, integer programming, mobile sensors, target coverage, wireless sensor network

## Abstract

We formulate and analyze a generic coverage optimization problem arising in wireless sensor networks with sensors of limited mobility. Given a set of targets to be covered and a set of mobile sensors, we seek a sensor dispatch algorithm maximizing the covered targets under the constraint that the maximal moving distance for each sensor is upper-bounded by a given threshold. We prove that the problem is NP-hard. Given its hardness, we devise four algorithms to solve it heuristically or approximately. Among the approximate algorithms, we first develop randomized (1−1/e)-optimal algorithm. We then employ a derandomization technique to devise a deterministic (1−1/e)-approximation algorithm. We also design a deterministic approximation algorithm with nearly ${▵}^{-1}$ approximation ratio by using a colouring technique, where ▵ denotes the maximal number of subsets covering the same target. Experiments are also conducted to validate the effectiveness of the algorithms in a variety of parameter settings.

## 1. Introduction

Wireless Sensor Networks (WSNs) are widely valued and have received significant attention in the last two decades. They are low cost and flexible thus they are widely applied in many applications scenarios ranging from health-care [1,2], industrial inspection [3], environmental monitoring [4,5], military defense [6,7], agriculture monitoring [8,9]. Coverage, as a fundamental issue in WSNs, has a direct impact on the networks’ efficiency. Based on the range of interesting, coverage problem was divided into area coverage [10,11], target coverage and barrier coverage [12,13]. In area coverage, each point in the entire 2D/3D range of interest (ROI) needs to be observed by at least one sensor. In target coverage, the objective is to ensure that a set of finite points located in the ROI are covered. Barrier coverage mainly focuses on detecting the intrusion across the borders of ROI. Most of the research on deployment problem focused on the area coverage. However, target coverage is also of primary importance as it is common in applications. For example, in information collection, only the information on certain points needs to be collected. As sensors may be randomly deployed in a large area, relocating a subset of mobile sensors is often required to ensure effective coverage over the monitored area. For battery-powered sensors, the energy consumption in movement is much higher than that in sensing and communication. Therefore, efficient sensor movement and scheduling strategies are called for to save energy while still meeting the coverage requirement or improve coverage quality with limited mobility [14,15,16].

In this paper, we study a target coverage problem with sensors of limited mobility in Mobile Wireless Sensor Network (MWSM). The network model was first presented in Reference [17]. In this network model, targets would be covered in a disk centred the sensor, that is, the sensor covers a target if it locates in the disk of the target. Each mobile sensor can cover more than one target if it locates in the overlapped region. The surveillance region is divided into several subareas, where a different subset of targets is detected/covered. Liao et al. [17] aim to minimize the sum of movement distances subject to covering all the targets. Considering there are not always enough sensors to cover all the targets and the energy of each sensor is limited, we want to study how to cover maximized number of targets with sensors of limited mobility. We call it Maximum Target Coverage with Limited Mobility (MTCLM) Problem. As far as we know, the problem is first presented in this paper. We prove that the MTCLM problem is NP-hard, and propose four algorithms, including one heuristic algorithm and three approximation algorithms. The heuristic algorithm is to choose an available subset at present that has the most increased profit. The approximation algorithms include a randomized (1−1/e) approximation algorithm, where *e* denotes the base of the natural logarithm; a deterministic (1−1/e) approximation algorithm by applying derandomiziation technique, and a deterministic ${▵}^{-1}$ approximation algorithm based on graph colouring technique, where ▵ is the maximal number of subsets covering the same target. Approximation algorithms are usually related to NP-hard problems. Since it is impossible to solve NP-hard in polynomial time, a polynomial-time suboptimal solution is acceptable. Different from heuristic algorithms, approximation algorithms can get a quality-guarantee solution, thus it requires provable solution quality and provable running time range.

To sum up, our contributions in this paper are the following:

1. We formulate the MTCLM problem and prove it NP-hard.
2. We propose four algorithms for the MTCLM problem, including one heuristic algorithm and three approximation algorithms.
3. We conduct experiments to validate the effectiveness of each algorithm in different conditions.

The rest of this paper is organized as follows—Section 2 reviews related works. The network model, the problem description and hardness are given in Section 3. Section 4 describes the four algorithms we present for the MTCLM problem. Section 5 presents the simulation results and investigates the performance of the algorithms proposed. Section 6 concludes the paper.

## 2. Related Works

Coverage Problem is so crucial that much research has done in the literature. Several survey papers related to the coverage issue in WSNs have been published [18,19,20,21,22], conclude the existing studies in different view. Reference [20] is the latest one published in 2019. It concluded the papers into three catalogues of coverage protocols: coverageaware deployment protocols, sleep scheduling protocols and cluster-based sleep scheduling protocols. It proposed that the more realistic model in WSNs is the future direction. Reference [22] described the deployment techniques in WSNs in detail, including Genetic Algorithms, Computational Geometry, Artificial Potential Fields, and Particle Swarm Optimization, and classifies the recent studies to the techniques. Reference [18] mainly concluded the studies on mobile wireless sensor networks (M-WSNs). It concluded that there were mainly four techniques to solve these problem proposed in M-WSNs, including optimization technique, computational geometry based technique, the virtual force-based technique, and geometry pattern based technique. Connectivity was usually considered in coverage problems [21]. Different from the above papers focusing on the deterministic model, Reference [19] concluded the coverage problem with uncertain properties and summarized the relevant models. Although the coverage problem had gotten so much attention, when a new technique was introduced to WSN, there were still considerable new researches. For example, with energy harvest technique getting mature, References [23,24,25,26,27,28] were proposed to study the coverage problem on the rechargeable WSN.

Most of the research on deployment problem focused on the area coverage. Target coverage is also an important topic. Most target coverage problems were NP-hard problem, optimization algorithms were applied to them including evolutionary algorithms [11,29,30,31,32] and combinatiorial algorithms [17,33,34,35,36].

A constrained Pareto-based multi-objective evolutionary approach (CPMEA) was proposed to find Pareto optimal layouts that maximized the coverage and minimized the sensors energy consumption while maintaining full connectivity between sensors [11]. A centralized genetic approach was provided to minimize the number of sensors and ensure targets *K*-coverage and *M*-connectivity simultaneously in Reference [29]. The fitness function was defined as a weighted sum of three factors: minimizing the number of sensors, maximizing the coverage performance and maximizing the communication connectivity. Dahiya et al. [30] proposed an algorithm to maximize the coverage of moving targets. They sampled the points on the trajectories of the mobile sensors uniformly to build a stationary probability distribution so that the uncertainty in the position of targets was fixed. A particle swarm intelligence (PSI)-based deployment algorithm was proposed to find the minimum number of static sensors to cover given targets in Reference [32]. Roselin et al. [31] proposed a sleep scheduling protocol to find disjoint covered set to extend the network lifetime while fulfilling the coverage and connectivity. Considering there were some crucial targets not be covered well, they classified sensors into four kinds and set their heuristic value, handled carefully the sensors which monitored the crucial targets.

The network model in our paper was first presented by Liao et al. [17]. In this paper, the Minimum Movement Target Coverage (MMTC) Problem was proved to be NP-hard. An extended Hungarian method was proposed to solve a particular case of the MMTC problem in which the distance between each pair of targets was longer than two times of sensing radius of sensors and a target based Voronoi greedy algorithm to solve the general case of MMTC. Another heuristic solution by minimizing the number of sensors needed was proposed in Reference [33]. Besides that, network connectivity was also considered in this paper. Another special case of MMTC, *k*-sink MMTC problem was proposed by Chen et al. [34], in which the sensors were located at *k* base stations and each station had infinite sensors, and  a PTAS was proposed to obtain a (1+ϵ) approximation solution. Reference [35] studied the MMTC problem with restricted mobility that sensors could move only in two mutually perpendicular directions. A heuristic algorithm was proposed to ensure coverage and connectivity. Nguyen et al. [36] proposed a more general problem for the target coverage and network connectivity than the MMTC problem, termed the Maximum Weighted Target Coverage and Sensor Connectivity with Limited Mobile Sensors (TAR-CC) problem. In this paper, a maximum-coverage-based algorithm and a Steiner-treebased algorithm were proposed. In Reference [37], they also showed the hardness of some related topic on target coverage problem and rectified the incorrectness of the proof in Reference [17] by reducing MMTC problem to the minimum geometric disk cover problem. No approximation algorithm for the MMTC problem has been presented until now.

In our paper, we study the MTCLM problem to cover maximum number of targets with sensors with limited mobility, which is different from the Maximum Weighted Target Coverage problem in Reference [36] because the mobility is limited. And the maximumcoverage-based algorithm is not suitable anymore.

## 3. Preliminary and Problem Statement

### 3.1. Network Model and Problem Definition

**Figure 1:**
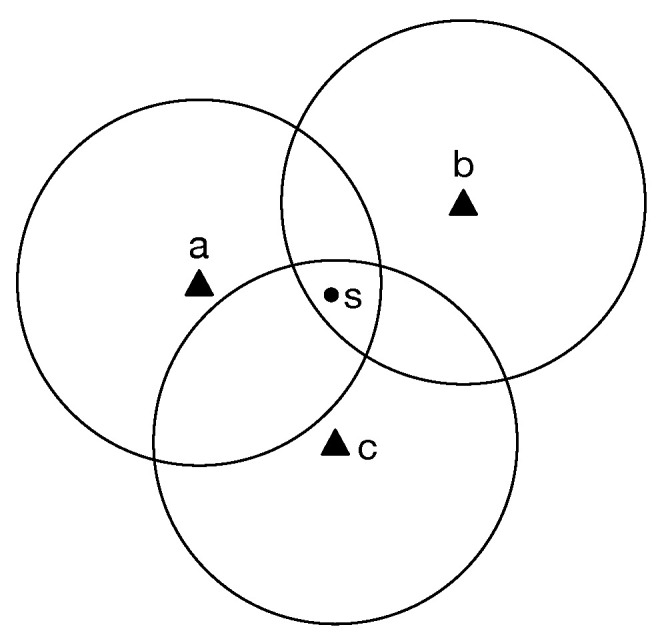
Subareas divided by targets.

We study the MTCLM problem in the following model. All the networking nodes are located in an obstacle-free surveillance region, including sensors and targets. The network is represented as N(T,S,D). $T=\{t_{1},t_{2},\ldots ,t_{N}\}$ is the set of *N* targets distributed uniformly and randomly, each of which has its known position. $S=\{s_{1},s_{2},\ldots ,s_{M}\}$ is a set of *M* homogeneous mobile sensors with the same sensing radius *r*, which are supposed to schedule to cover all the targets. The disk model is adopted. That means a target *t* is said to be covered if and only if at least one mobile sensor’s final position is in the disk centred target *t* with radius *r*. The mobile sensors have known initial position, and they can move in any direction and stop anywhere. In Figure 1, the mobile sensor *s* can cover target *a*, *b*, *c* because *s* is in the disk O(a), O(b),O(c) at the same time, where O(t) is a disk centred a target *t* with radius *r*. As a result, the surveillance region can be divided into several subareas. In some subarea, a subset of targets is covered. Let U⊆T denote a subset of targets, R(U) be the corresponding subarea where the sensors cover the subset U of targets. Let $\mathrm{SU}=\{U|U\subseteq T\;and\;R\left(U\right)={⋂}_{t\in U}R\left(t\right)\}$ denote the set of subsets corresponding to the subareas in surveillance region. Let *K* denote the number of the subareas/subsets, that is, |SU|=K.

Considering the limited energy of each mobile sensor, we present a maximum target coverage problem with sensors of limited distance. Let $D=\{d_{1},d_{2},\ldots ,d_{M}\}$ be the mobile sensors’ corresponding moving distance constraint. The maximum target coverage problem with sensors of limited mobility is defined below:

**Definition** **1.** *Maximum Target Coverage with Limit Mobility (MTCLM) Problem: Given M mobile sensors and N targets located at surveillance domain with known positions, the mobile sensor are homogeneous with the same sensing radius. With the maximal moving distance of sensors constrained, to find how to schedule the sensors to cover as many targets as possible.*

Table 1 summarizes the notations used in this paper.

### 3.2. Hardness of The Problem

We prove the MTCLM problem is NP-hard by reducing the minimum geometric disk cover (MGDC) problem to it as in Reference [37]. The MGDC problem is NP-hard and its definition is showed as following:

**Definition** **2.** *The minimum geometric disk cover problem (MGDC) [38]: Given a set of m points $P=\{p_{1},p_{2},\ldots ,p_{m}\}$, a disk radius r, and a constant $k\in Z^{+}$, does there exist a set of centers $C=\{c_{1},c_{2},\ldots ,c_{n}\}$ such that every point in P is covered by a disk centred at one of the centers in C and the cardinality of C, that is, n is not greater than k?*

Then we prove the MTCLM problem is NP-hard.

**Theorem** **1.** *The MTCLM problem is NP-hard.*

**Proof.**  We consider a special case of the MTCLM problem when the maximal distance of each mobile sensors is not constrained, that is, $d_{i}=\infty$ for $d_{i}\in D$, each mobile sensor alway be able to reach any subarea to cover targets. By reducing the MGDC problem to the special case of the MTCLM problem, we prove the MTCLM problem is NP-hard.

Given an MGDC instance like Definition 2, we construct an MTCLM instance. In this instance, there are M=n mobile sensors and N=m targets; the sensing radius of mobile sensors is *r*. The set of centers $C=\{c_{1},c_{2},\ldots ,c_{n}\}$ is the set of final positions of the mobile sensors. If the MGDC instance is satisfied, that is, there exist n≤k=M disks covering all the points P, there must be less than *M* sensors located in centers $C=\{c_{1},c_{2},\ldots ,c_{n}\}$ and the maximum number of covered targets in the MTCLM problem is *N*. Conversely, if the maximum number of targets covered in the MTCLM instance is *m*, there exist n≤k centers to cover all the points in P. The MGDC instance is satisfied. Else if the maximum number of targets covered in the MTCLM instance is less than *m*, there does not exist n≤k centers all the points in P. The MGDC instance is not satisfied.

Thus, the MTCLM problem is NP-hard. □

## 4. Algorithms

In this section, we propose four algorithms for the MTCLM problem, a heuristic algorithm, a randomized approximation algorithm, a derandomized approximation algorithm, and a deterministic approximation algorithm, respectively. Given an MTCLM instance, we should first find the subareas and its corresponding subsets, then find the minimal distances $\omega_{ij}$ between each mobile sensor *i* and each subarea *j*. We will use the distance algorithm proposed in Reference [34] to solve the above two problems. That will take $O(MN^{2})$ time. After pretreatment, we focus on the core issue on how to schedule the mobile sensors to cover the targets.

With the moving distance constraint, sensors can reach only some subareas. We construct a bipartite graph G≜((S,JU),E). Each vertex i∈S denotes a sensor in S. Each vertex j∈JU denotes a subarea covering a set of targets $U_{j}\in \mathrm{SU}$. When the distance between sensor *i* and subarea $R(U_{j})$ is smaller than the constrained moving distance of sensor *i*, that is, $\omega_{ij}\leq d_{i}$, there is an edge between vertex *i* and *j*, where $\omega_{ij}$ is the minimum distance between sensor *i* and subarea *j* obtained in the pretreatment. The MTCLM problem is turning to be a matching problem except the weight is submodular. We formulate the MTCLM problem by an integer linear program (ILP) as following:

(1) $$\begin{array}{cc} \max & \sum_{t\in T}y_{t}, \end{array}$$

(2) $$\begin{array}{ccc} s.t. & \sum_{j\in \mathrm{JU}}x_{ij}\leq 1 & for\;every\;sensor\;i\in S, \end{array}$$

(3) $$\begin{array}{ccc} \; & \sum_{i\in S}\sum_{j:t\in U_{j},\left(i,j\right)\in E}x_{ij}\geq y_{t} & for\;every\;target\;t\in T, \end{array}$$

(4) $$\begin{array}{ccc} \; & x_{ij}\in \left\{0,1\right\} & i\in S,j\in \mathrm{JU}, \end{array}$$

(5) $$\begin{array}{ccc} \; & y_{t}\in \left\{0,1\right\} & t\in T, \end{array}$$

where $y_{t}\in \left\{0,1\right\}$ denotes if target t∈T covered or not. $x_{ij}\in \left\{0,1\right\}$ denotes if sensor i∈S is scheduled to subarea $R(U_{j})$, $U_{j}\in \mathrm{SU}$. The first constraint is the feasibility constraint, the second is the coverage constraint.

### 4.1. Greedy Algorithm

In this subsection, we propose a heuristic algorithm by choosing the available subset which contains the most uncovered targets for each sensor.

In Algorithm 1, let $X=\{x_{ij}:\left(i,j\right)\in E,x_{ij}\in \left\{0,1\right\}\}$ denote the solution. Let ${\hat{U}}_{j}\subseteq U_{j}$ denote the uncovered targets in current iteration, ∀j∈JU. Let CT⊆T denote the covered targets. For each sensor i∈S, to find the accessbile subset with the most uncovered targets. The time complexity of this algorithm is O(M*N).
**Algorithm 1:** MTCLM_GREEDY(*G*, SU).Input:graph *G*, SUOutput:*X*,CT.1: $X=\{x_{ij}\leftarrow 0,\;\forall \left(i,j\right)\in E\}$;2: CT←∅;3: ${\hat{U}}_{j}\leftarrow U_{j},\;\forall j\in \mathrm{JU}$;4: **for each** i∈S **do**5:  $l\leftarrow arg\;max{}_{j\in \mathrm{JU},(i,j)\in E}\left|{\hat{U}}_{j}\right|$;6:  $x_{il}=1$;7:  $\mathrm{CT}\leftarrow \mathrm{CT}⋃U_{l}$;8:  $\hat{U_{j}}\leftarrow \hat{U_{j}}\setminus U_{l},\;\forall j\in \mathrm{JU}$;9: **end for**10:**return**X,CT;

### 4.2. Randomized Algorithm

The Greedy algorithm presented above can not guarantee algorithm’s performance. Thus, we present three approximation algorithms which can make sure that the deviation of approximate solutions from the optimal value would not exceed a certain range. In this subsection, we propose a randomized approximation algorithm to obtain the expected value of the solution relative to the optimal value.

In this algorithm, we first obtain the optimal solution, $\left\{y_{t}^{*}\right\}$ and {$x_{ij}^{*}$}, of the Linear Program Relaxation (LPR) of formulas ILP, by replacing the constraints (4), (5) with $x_{ij}\in \left[0,1\right]$ and $y_{t}\in \left[0,1\right]$. Then for each vertex i∈S, we choose edge (i,j) with prob. $x_{ij}^{*}$. Without changing the approximation ratio, we shift the covered targets by avoiding to cover the same subset. We run the algorithm for several times until we obtain the acceptable approximation solution.

**Theorem** **2.** *Algorithm 2 is a randomized (1−1/e)-approximation algorithm for the MTCLM problem.*

**Proof.**  In this algorithm, the prob. for sensor i∈S covering target t∈T is $\sum_{j:t\in U_{j},\left(i,j\right)\in E}x_{ij}^{*}$ and each sensor i∈S sends to cover subsets independently. Therefore, the overall prob. of not covering target *t* sums up to $\prod_{i:(i,j)\in E}\left(1-\sum_{j:t\in U_{j}}x_{ij}^{*}\right)$. According to Arithmeticgeometric mean inequality and the coverage constraint of ILP, we can show that

$$\prod_{i\in S:(i,j)\in E}\left(1-\sum_{j:t\in U_{j}}x_{ij}^{*}\right)\leq {\left(1-y_{t}^{*}/m\right)}^{m},$$

where *m* denotes the maximal degree of any vertex in SU in graph *G*. Again, it holds algebraically that $1-{\left(1-y_{t}^{*}/m\right)}^{m}\geq y_{t}^{*}*\left(1-1/e\right)$. It then follows that the prob. of covering target *t* in the algorithm is

$$1-\prod_{i\in S:(i,j)\in E}\left(1-\sum_{j:t\in U_{j}}x_{ij}^{*}\right)\geq y_{t}^{*}*\left(1-1/e\right),$$

Remind that $\sum_{t\in T}y_{t}^{*}$ is the optimal value of LPR, bigger than the optimal value of ILP. Let OPT denote the optimal value of ILP. Then the expected number of covered targets is

$$\sum_{t\in T}\left(1-\prod_{i\in S:(i,j)\in E}\left(1-\sum_{j:t\in U_{j}}x_{ij}^{*}\right)\right)\geq OPT*\left(1-1/e\right).$$

It holds that the randomized rounding algorithm gives a randomized (1−1/e)-optimal solution.

Linear programs can be solved in polynomial time and so is Algorithm 2. The theorem is proved. □

In Algorithm 2, we use the recycle variable max_round to avoid the sensors covering the same subset that would improve results and reduce instability in experiments.
**Algorithm 2:** MTCLM_RANDOM (*G*, SU).Input:graph *G*, SUOutput:*X*,CT.1:$X=\{x_{ij}\leftarrow 0:\forall i\in S,j\in \mathrm{JU}\}$;2:CT←∅;3:computer the optimal solution to the LP $x^{*}$,$y^{*}$;4:**for each**i∈S**do**5:  set max_round a positive constant.6:  **while** max_round>0 **do**7:   set $x_{ij}=1$ with probability $x_{ij}^{*}$;8:   **if** $x_{kj}=0\;\forall \;k\in \left[1,i-1\right]$ **then**9:    set $x_{ij}=1$;10:    $\mathrm{CT}=\mathrm{CT}⋃U_{j}$
11:    break;12:   **else**
13:    set $x_{ij}=0$14:    max_round=max_round−1;15:   **end if**
16:  **end while**
17:**end for**18:**return***X*,CT;

The method of conditional expectation can be used to derandomize the solution, but the computing complexity depends on the maximal degree *n* of any vertex i∈S in graph *G*, that is, the maximal number of subareas a sensor can reach. We show the derandomized algorithm as below: Let $J_{i}$ denote the set of vertices {j:(i,j)∈E} for sensor *i*. $\bar{j}=\left\{q\in \mathrm{JU},\;q\neq j\right\}$. In each iteration *k*, $X_{k-1}=\left\{x_{ij}|i\in \left[1,k-1\right],j\in \mathrm{JU}\right\}$ is fixed. Set $x_{kq}=1$ to let the current conditional expectation maximized, that is, $q=argmax_{i\in J_{k}}E\left[S_{L}|x_{ki}=1;x_{k\bar{i}}=0;X_{k-1}\right]$, where $S_{L}$ denotes the value of the LPR. After *M* iterations, we can obtain deterministic (1−1/e)-optimal solution. In this algorithm, O(Mn) linear programs need to be solved. At the worst cast, n=K is the number of vertices in JU. The number of subareas $K\leq 4N^{2}$ was proved in Reference [34]. Thus, $n=O(N^{2})$ at the worst case. That would make the time complexity of the derandomized algorithm too high. Even though, there are still lots of situations in which *n* is constant in practical. With the moving distance constraint, each sensor is only allowed to reach a constant subarea, that is the derandomized algorithm suitable for.

**Theorem** **3.** *Algorithm 3 is a deterministic (1−1/e)-approximation algorithm when n is constant.*

**Proof.**  As the explanation above, we prove the theorem by induction.

When k=0, $E_{0}=E\left[S_{L}\right]$ with no $x_{ij}$ is fixed, is the expectation of the total number of covered targets. $E_{0}\geq \left(1-1/e\right)*OPT$ is proved in Theorem 2, where OPT is the optimal solution of the ILP.

In each iteration *k*, $E_{k-1}=E\left[S_{L}|X_{k-1}\right]$ where $X_{k-1}$ is fixed. And $E_{k-1}=\sum_{j\in J_{k}}E\left[S_{L}|x_{kj}=1,x_{k\bar{j}}=0;X_{k-1}\right]p\left(x_{kj}=1,x_{k\bar{j}}=0\right)$. We choose $q=argmax_{i\in J_{k}}E\left[S_{L}|x_{ki}=1;x_{k\bar{i}}=0;X_{k-1}\right]$, thus $E_{k}=E\left[S_{L}|x_{kq}=1,x_{k\bar{q}}=0;X_{k-1}\right]\geq E_{k-1}$.

After *M* iterations, the number of covered targets is $E_{M}\geq E_{0}\geq \left(1-1/e\right)*OPT$.

To sum up, there are *M* iterations, and in each iteration, we need to solve at most *n* linear programs. Thus the time complexity is O(MnL), assuming the time complexity of a linear program is O(L). When each sensor is only allowed to reach a constant subarea, that is, *n* is a constant, Algorithm 3 is linearly solvable. Now, we prove that Algorithm 3 is a deterministic (1−1/e)-approximation algorithm. □

**Algorithm 3:** MTCLM_DERANDOMIZED(*G*, SU).
Input:graph *G*, SUOutput:*X*,CT.1:$X=\{x_{ij}\leftarrow 0:\forall i\in S,j\in \mathrm{JU}\}$;2:CT←∅;3:computer the optimal solution to the LP: $x^{*}$,$y^{*}$;4:
**for each** i∈S **do**
5:  $q=argmax_{j\in J_{i}}E\left[S_{L}|x_{kj}=1,x_{k\bar{j}}=0;X_{k-1}\right]$;6:  set $x_{iq}=1$, $x_{i\bar{q}}=0$;7:  $\mathrm{CT}=\mathrm{CT}⋃U_{q}$;8:
**end for**
9:**return**X,CT;


### 4.3. Deterministic Algorithm

The randomized algorithm needs to run several times to reduce instability. The derandomized algorithm can obtain an approximation solution determinately but costs too much time complexity at most time. In this subsection, we propose a deterministic algorithm to obtain a nearly ${▵}^{-1}$ approximation value with less time complexity, where Δ is the maximal number of subsets covering the same target. Let $\left\{y_{t}^{*}|t\in T\right\}$ and $\left\{x_{ij}^{*}|\left(i,j\right)\in E\right\}$ denote the optimal solution of the LPR. We round up each of them to the closest fraction of the form a/H, *H* is a large integer and *a* is an integer between 0 and *H*. Mathematically, let ${\hat{y}}_{t}≜\left\lceil y_{t}^{*}H\right\rceil /H$ and ${\hat{x}}_{ij}$ is defined similarly. (The rounding would incur a quantization error which we analysis later). For the graph G≜((S,JU),E), we duplicate each node j∈JU to $H{\hat{x}}_{ij}$ identical nodes covering the same set of targets and connect each duplicated node to the neighbor of *j*. We call the new graph the auxiliary graph $\bar{G}$. We then find an edge-colouring of $\bar{G}$ such that any pair of edge sharing the same vertex in S is coloured differently. We can prove that *H* colours are sufficient. We can show by pigeon-hole principle that we can always find a colour that the vertices in JU covered by the edges in the colour cover at least $\sum_{t\in T}y_{t}^{*}/\Delta$ targets, that is, $\sum_{i\in S}\sum_{j\in \mathrm{JU}:(i,j)\in E}{\hat{x}}_{ij}\geq \sum_{t\in T}y_{t}^{*}/▵$. To prove this by contradiction, assuming that this is not true. Then the

$$\sum_{i\in S}\sum_{t\in T}\sum_{j:t\in U_{j},\left(i,j\right)\in E}{\hat{x}}_{ij}\leq ▵\sum_{i\in S}\sum_{j\in \mathrm{JU}:(i,j)\in E}{\hat{x}}_{ij}\leq \sum_{t\in T}y_{t}^{*}.$$

It is leading to contradiction with the constraint (3) of the ILP.

The above analysis immediately implies that selecting the edges covered by the best coloured induced a $\Delta^{-1}$-optimal solution. More applications of the method can be seen in Reference [39].

Each $y_{t}$ increases by at most 1/H by the rounding, hence increases the objective function by at most N/H. Taking quantization error into consideration, the approximation ratio is $\left(1-\frac{N}{H}\right){▵}^{-1}$. The auxiliary graph $\bar{G}$ has at most M+HN nodes, M*HN edges, to find a proper coloration by greedy will take time O(MHN). If we set $H=N^{2}$, the approximation ratio is $\left(1-1/N\right){▵}^{-1}$ and the time complexity is $O(MN^{3})$. Thus we get the Theorem 4.

**Theorem** **4.** *Algorithm 4 is a nearly ${▵}^{-1}$ approximation algorithm*

**Algorithm 4:** MTCLM_COLOUR (*G*, SU).
Input:graph *G*, SUOutput:*X*,CT.1:$X=\{x_{ij}\leftarrow 0:\forall i\in S,j\in \mathrm{JU}\}$;2:CT←∅;3:computer the optimal solution to the LP: $x^{*}$,$y^{*}$;4:${\hat{y}}_{t}≜\left\lceil y_{t}^{*}H\right\rceil /H$;5:${\hat{x}}_{ij}≜\left\lceil x_{ij}^{*}H\right\rceil /H$;6:build auxiliary graph $\bar{G}$;7:obtain a legal colouring of $\bar{G}$ greedily;8:
**for** k∈[1,H] **do**
9:  calculate the number of targets covered by each colour *k*;10:
**end for**
11:obtain colour *q* with the maximum number of covered targets;12:obtain the edges $E_{q}$ coloured by *q* in graph $\bar{G}$;13:
**for** $\left(i,j^{\prime }\right)\in E_{q}$ **do**
14:  **if**$j^{\prime }$ is the duplication nodes of *j* in graph *G* **then**15:   set $x_{ij}=1$;16:   $\mathrm{CT}=\mathrm{CT}⋃U_{j}$;17:  **end if**
18:
**end for**
19:**return**X,CT;


## 5. Simulation Experiments

**Figure 2:**
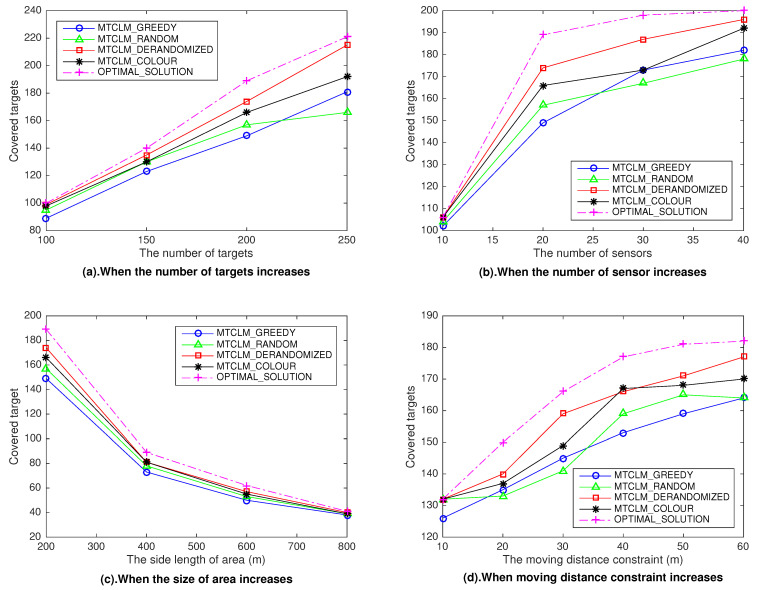
To show the impression of four parameters on the four algorithms and the optimal solution. (**a**) shows the impression of the number of targets; (**b**) shows the impression of the number of sensors; (**c**) shows the impression of size of area; (**d**) shows the impression of the moving distance constraint of sensors.

Even though the performance of some algorithms we proposed have been proved in theory, we still conduct a set of simulation experiments by using Matlab to compare them, validate their effectiveness, and show the impression of some parameters. We consider four network parameters that may impact on the number of covered targets: the number of targets *M*, the number of sensors *N*, the size of the surveillance region, the moving distance constraint D. In the experiments, there are 200 targets and 20 mobile sensors uniformly, randomly generated in a square region of size 200 m × 200 m. The sensors’ coverage radius is r=20 m, and their moving distance constraint is 40 m. Even though the moving distance constraint of each sensor can be different, in our experiments, we assume they are the same. To test the impression of each parameter, we vary it in experiments and keep the other parameters keep the same. For each combination of network parameters, we randomly generate ten instances of the network and report the mean performance result. We show the results in Figure 2 below. In Figure 2, we compute the results of the algorithms we present and compare them to the optimal solutions. The optimal solutions can be obtained by using Matlab’s toolbox ’Yalmip’, which is a free optimization solution tool developed by Lofberg.

In Figure 2a, the number of targets varied from 100 to 250. In Figure 2b, four different numbers of sensors are considered, namely N=10,20,30,40. We can observe that, when the number of targets increases or the number of sensors increases, the number of targets covered increases.

In Figure 2c, four different sizes of area are considered, namely 200 m × 200 m, 400 m × 400 m, 600 m × 600 m, 800 m × 800 m. We observe that when the size of the surveillance regionincreases, the number of targets covered decreases. It is easy to understand that when the size of the surveillance region increases whereas the number of targets remain unchange, the average density of targets gets low. The average number of targets in a subset decrease hence the total number of covered targets decreases.

In Figure 2d, the moving distance constraint varied from 10 to 60. It is observed that when the moving distance constraint increases, the number of targets covered increase, but the growth rate would become slower.

In the simulation experiments, we obtain the numbers of maximum covered tagets through four algorithms, which are smaller than the optimal solutions. We calculate the lower bound of the performance of each algorithm, which is the minimum ratio of the algorithm solution to the optimal solution in the experimental data. Table 2 lists the lower performance bound of each algorithm:

The approximation ratio is a performance lower bound, for example, in a maximization problem, approximation ratio α of the algorithm indicates the approximate solution obtained by the algorithm would at least α times greater than the optimal solution. Remind that the approximation ratio of algorithm MTCLM_RANDOM and MTCLM_DERANDOMIZED is 1−1/e≈0.63, the approximation ratio of algorithm MTCLM_COLOUR is ${▵}^{-1}\leq 0.5$ which depends on the parameter ▵, where ▵ denotes the maximal number of subsets covering the same target. As shown in Table 2, the experimental lower bounds of the performance of the algorithms meet the theoretical analysis. Also, we can see that MTCLM_GREEDY algorithm achieves relatively worse results especially when the size of area is small and the number of targets and sensors remains unchange. That is because when the density of the target and the sensor becomes higher, it cannot just greedily select the target area, it needs to rely on global information. The performance of algorithm MTCLM_RANDOM is with a certain degree of randomness. The algorithm MTCLM_DERANDOMIZED has the best performance but it has higher time complexity than others. The algorithm MTCLM_COLOUR performs better than the theoretical analysis. There are two possibilities, one is that the example with the worst performance is not found, and the other is that there is a more accurate approximation analysis method for algorithm MTCLM_COLOUR, which can be studied further.

Combined with theoretical analysis, we know how to choose a suitable algorithm among these four algorithms according to the actual situation. When the accuracy requirements are not very high and the time requirements are very strict, the MTCLM_GREEDY algorithm and the MTCLM_RANDOM algorithm are good choices. Because these two algorithms only need linear time complexity, but the MTCLM_GREEDY algorithm cannot get a provable performance and the MTCLM_RANDOM has a randomly biased performance. When the target density is not large, that is, the parameter ▵ is small, the MTCLM_COLOUR algorithm will achieve good performance. When a sensor can only reach a constant number of target areas, that is, the parameter *n* denoting the number of target areas a sensor can reach is a constant, the time complexity of the MTCLM_DERANDOM algorithm is not high, and a good approximation ratio can be obtained.

## 6. Discussion

In this paper, we are the first to present the MTCLM problem to maximize the number of covered targets with sensors under limited mobility constraints. It applies to improving the utilization efficiency of insufficient sensors. Considering that the MTCLM problem is NP-hard and the requirements for time and performance are different in actual situations, we have proposed four algorithms and provided proof of algorithm performance for three of them. Through theoretical and experimental analysis, we also provide the direction of the algorithm selection in actual situations. Moreover, the proposed solutions are applicable to solving relevant resource allocation problems with the same model. In the future, it is interesting to find a more efficient algorithm with a better approximation ratio.

## Figures and Tables

**Table 1:** Notations.

Symbol	Definition
T	the set of targets: $\left\{t_{1},t_{2},\ldots ,t_{N}\right\}$
*S*	the set of sensors: $\left\{s_{1},s_{2},\ldots ,s_{M}\right\}$
D	the distance constraint of sensors: $\left\{d_{1},d_{2},\ldots ,d_{M}\right\}$
U	a subset of sensors: U⊆T
SU	the set of subsets: $\left\{U_{1},U_{2},\ldots ,U_{K}\right\}$
*M*	the number of sensors: |S|
*N*	the number of targets: |T|
*K*	the number of subareas: |SU|
*r*	the sensing radius of sensors
R(t)	the sensing region of target *t*
R(U)	the intersection of R(t) for all t∈U

**Table 2:** The experimental lower bounds of the performance of the algorithms.

Name of the Algorithm	Lower Bound of the Performance
MTCLM_GREEDY	0.78
MTCLM_RANDOM	0.75
MTCLM_DERANCOMIZED	0.91
MTCLM_COLOUR	0.86
